# Oxidative Addition of Dihydrogen to Divanadium in Solid Ne: Multiple‐Bonded Triplet HVVH and Singlet V_2_(μ‐H)_2_

**DOI:** 10.1002/anie.202004241

**Published:** 2020-05-18

**Authors:** Olaf Hübner, Hans‐Jörg Himmel

**Affiliations:** ^1^ Anorganisch-Chemisches Institut Universität Heidelberg Im Neuenheimer Feld 270 69120 Heidelberg Germany

**Keywords:** hydrides, matrix isolation, MRCI calculations, multiple bonding, vanadium

## Abstract

Dinuclear compounds of early transition metals with a high metal–metal bond order are of fundamental interest due to their intriguing bonding situation and of practical interest because of their potential involvement in catalytic processes. In this work, two isomers of V_2_H_2_ have been generated in solid Ne by the reaction between V_2_ and H_2_ and detected by infrared spectroscopy: the linear HVVH molecule (^3^Σ_g_^−^ ground state), which is the product of the spin‐allowed reaction between V_2_ (^3^Σ_g_^−^ ground state) and H_2_, and a lower‐energy, folded V_2_(μ‐H)_2_ isomer (^1^A_1_ ground state) with two bridging hydrogen atoms. Both isomers are characterized by metal–metal bonding with a high bond order; the orbital occupations point to quadruple bonding. Irradiation with ultraviolet light induces the transformation of linear HVVH to folded V_2_(μ‐H)_2_, whereas irradiation with visible light initiates the reverse reaction.

## Graphical abstract

**Into the fold**: Linear HVVH and folded V_2_(μ‐H)_2_ have been generated in solid Ne and were detected by infrared spectroscopy. Both molecules are characterized by multiple metal–metal bonding with a high bond order.

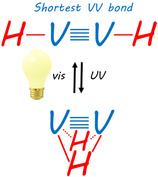

## Introduction

Many dinuclear complexes of early transition metals have been synthesized in which there are M_2_ units that show metal‐metal multiple bonding.^1^ Due to their electronic properties, such complexes show a unique chemical reactivity that makes them suitable for involvement in catalysis.^2^ Among these complexes, there are paddle‐wheel complexes like V_2_(DPhF)_4_ (DPhF=*N*,*N*‐diphenylformamidinate), showing short V−V distances of less than 200 pm with a triple bond between the metal atoms.^3^, ^4^ To stabilize complexes with a strong bonding between the metal atoms, it is advisable to reduce the number of ligands as far as possible. However, due to the increased reactivity of the low‐coordinated metals in such complexes, it is necessary to use bulky ligands that shield the metal core. The prime example is the dinuclear chromium complex RCrCrR (R=C_6_H_3_‐2,6(C_6_H_3_‐2,6‐^*i*^Pr_2_)_2_) with a minimum number of ligands in a *trans*‐bent structure, which even shows quintuple bonding between the two chromium centers with a Cr−Cr distance of only 183.5 pm.^5^, ^6^ The electronic structure of HCrCrH and some other small model systems for this complex have been analyzed theoretically to confirm the presence of fivefold bonding and to understand the origin of the *trans*‐bent alignment.^7^, ^8^ However, in general, it is not clear to what extend the bulky ligands determine the structure. Therefore, it is in particular desirable to characterize, if possible, experimentally, systems like the prototypical HMMH molecules with terminally bonded hydrogen atoms and multiple metal–metal bonding. The matrix‐isolation technique is a versatile means to stabilize and investigate such molecules by isolating them in noble‐gas matrices.

An obvious way to generate HMMH molecules in inert‐gas matrices is the reaction of matrix‐isolated bare metal dimers with H_2_ in noble‐gas matrices. A larger number of bare transition‐metal dimers has been generated in noble‐gas matrices,^9^ and some studies showed that metal dimers exhibit a special reactivity. As to matrix isolation, an outstanding example is the reactivity of the titanium dimer. Ti_2_ reacts with dinitrogen by formation of cyclic Ti_2_(μ‐N)_2_, breaking even the strong triple bond, whereas Ti atoms do not show a corresponding reaction.^10^ Another example for the special reactivity of dimers is the platinum dimer cation Pt_2_^+^, which was shown to be able to activate ammonia in the gas phase (Pt_2_^+^+NH_3_→Pt_2_NH^+^+H_2_), whereas Pt^+^, Pt_3_^+^, and Pt_4_^+^ were not.^11^ However, work concerning the reaction of neutral transition‐metal dimers with dihydrogen is scarce. There are many studies in which the reaction of evaporated transition metals M with H_2_ has been investigated in matrices.^12^ Some of them also report products with the composition M_2_H_2_. The late‐transition‐metal compounds Rh_2_H_2_ 13 and Pd_2_H_2_ 14 have been reported to feature rhombic structures (bridging hydrogens), while the compounds HZnZnH, HCdCdH,^15^ and HHgHgH^16^ show linear structures. Furthermore, absorptions have been tentatively assigned to Mn_2_H_2_,^17^ HTiTiH,^18^ HZrZrH,^19^ and HCrCrH,^20^ and the dihydrogen complexes PdPd(H_2_)^14^ and PtPt(H_2_)^21^ have also been observed.

In this work, we report on the generation and characterization of matrix‐isolated V_2_H_2_ molecules. So far, the reaction of V_2_ with H_2_ has not been reported, only V atoms have been reacted with dihydrogen. Thermally evaporated V atoms in Kr and Ar matrices have been found to react with H_2_ only upon irradiation (320 nm<*λ*<380 nm) by formation of VH_2_, whereas V atoms in their electronic ground state do not react with H_2_.^22^ V atoms generated by laser ablation, however, react with H_2_ to give VH_2_ in Ne and Ar matrices also without specific irradiation.^23^

Also, for some transition‐metal‐cluster cations, among them the dimer cations, the reactivity with respect to D_2_ has been studied,^24^ and it was observed that V_2_^+^ reacts in the gas phase with D_2_ to yield V_2_D^+^.^25^ Interestingly, very recently, the reaction of V_2_^+^ with CO_2_ to yield V_2_O_2_^+^ has been investigated, and the surprising observation has been made that the intermediate V_2_O^+^ reacts considerably faster than V_2_^+^.^26^

**Figure 1:**
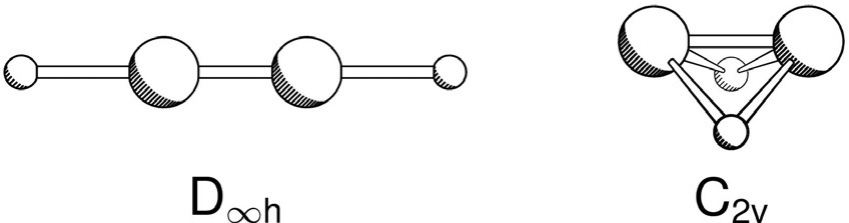
Structures of HVVH (left) and V_2_(μ‐H)_2_ (right).

In this work, it is shown by IR spectroscopy that neutral V_2_ reacts with H_2_ without a significant reaction barrier to linear HVVH under preservation of the spin multiplicity and to the V_2_(μ‐H)_2_ isomer with a nonplanar, folded structure (see Figure 1). The latter is generated, in particular, upon irradiation with light of a wavelength in the ultraviolet range between 250 and 385 nm.

To assist in the assignment of the observed bands and to obtain information on the electronic structure of the products, quantum‐chemical calculations are performed. Information on the energy and nature of the electronic ground states and low‐lying excited states is obtained by multireference configuration‐interaction (MRCI) calculations based on complete‐active‐space self‐consistent‐field orbitals. Vibrational frequencies are calculated by density‐functional calculations.

## Results and Discussion

### IR Spectra

**Figure 2:**
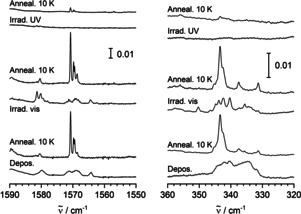
IR spectra at around 1570 and 340 cm^−1^ of matrices obtained by deposition of V and H_2_ in Ne, a) after deposition, b) after annealing to 10 K, c) after irradiation with visible light, d) after further annealing to 10 K, e) after irradiation with UV light, f) after another annealing to 10 K.

The IR spectra recorded after co‐depositing vanadium atoms and a mixture of Ne and dihydrogen show broader absorptions at around 1580, 1571, 1569, and 1564 cm^−1^, and furthermore absorptions in the region between 331 and 346 cm^−1^ that belong to the same compound (see Figure 2). Upon annealing to 10 K, four sharper bands appear at 1570.7 (strong), 1569.8 (medium), 1569.4 (medium), and 1568.6 cm^−1^ (weak), as well as a band in the low‐frequency regime at 343.4 cm^−1^ with a shoulder at 342.5 cm^−1^ at the expense of the broader bands. Upon irradiation with visible light, the broader bands re‐appear with a slightly changed intensity pattern; there is more intensity at around 1580 cm^−1^ with two maxima at 1581.4 and 1580.2 cm^−1^, and in the low‐frequency region, there are two pronounced absorptions at 342.4 and 340.3 cm^−1^. Upon renewed annealing to 10 K, the sharper bands that appeared in the first annealing again recover intensity at the expense of the broader bands. Upon irradiation with ultraviolet (UV) light, all those bands are extinguished, and upon further annealing to 10 K, the sharp bands only very weakly re‐appear.

**Figure 3:**
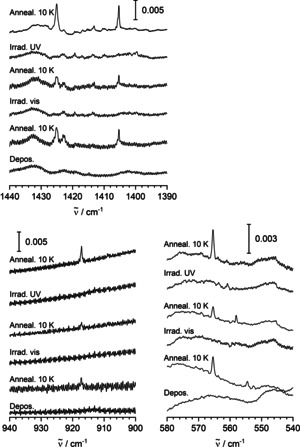
IR spectra at around 1420, 920, and 560 cm^−1^ of matrices obtained by deposition of V and H_2_ in Ne, a) after deposition, b) after annealing to 10 K, c) after irradiation with visible light, d) after further annealing to 10 K, e) after irradiation with UV light, f) after another annealing to 10 K.

Another set of absorptions is observed essentially only after annealing (to 10 K), but neither directly after deposition nor directly after irradiation with visible light. These absorptions are found at 1425.1, 1405.3, 917.2, and 565.4 cm^−1^ (see Figure 3). The bands clearly appear upon annealing after deposition, they appear less strongly upon annealing after irradiation with visible light, and they appear most strongly upon annealing after irradiation with UV light. At least after the irradiation with UV light, there are weaker absorptions red‐shifted with respect to the clear bands observed after annealing.

**Figure 4:**
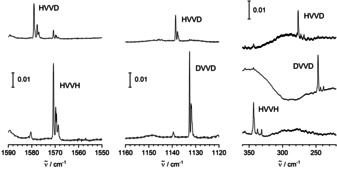
IR spectra at around 1570, 1140, and 300 cm^−1^ of matrices obtained by deposition of V and H_2_, D_2_, or HD in Ne after irradiation with visible light and annealing to 10 K.

For all those bands, corresponding counterparts are observed using D_2_ or HD. For the first set of absorptions, there are D_2_ counterparts of the four sharper signals that appeared after annealing to 10 K at 1132.6, 1131.9, 1131.8, and 1131.5(sh) cm^−1^, and counterparts in the low‐frequency regime at 247.0 and 246.2(sh) cm^−1^ (see Figure 4). Using HD, one group of counterparts is found at 1579.0, 1678.6(sh), 1577.7, and 1577.0 cm^−1^ near the absorptions found using H_2_, and another group of counterparts is found at 1138.6, 1138.3(sh), 1137.8, and 1137.4(sh) cm^−1^ near the absorptions found with D_2_. In the low‐frequency region, the HD counterparts are observed at 276.7 and 275.9(sh) cm^−1^. Furthermore, using HD, there appears another weaker band at 442.8 cm^−1^ not found in the experiments with H_2_ or D_2_.

**Figure 5:**
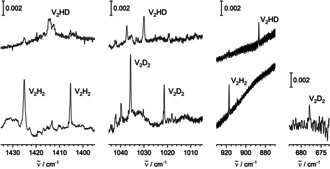
IR spectra at around 1420, 1030, 900, and 680 cm^−1^ of matrices obtained by deposition of V and H_2_, D_2_, or HD in Ne after irradiation with UV light and annealing to 10 K.

**Figure 6:**
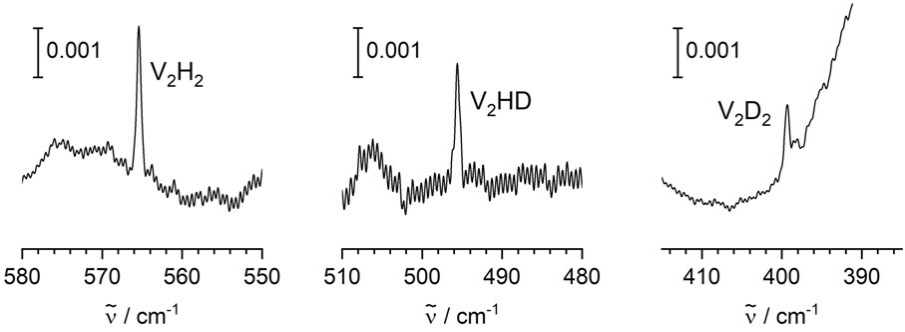
IR spectra at around 570, 490, and 400 cm^−1^ of matrices obtained by deposition of V and H_2_, D_2_, or HD in Ne after irradiation with UV light and annealing to 10 K.

For the second set of absorptions, the D_2_ counterparts are found at 1035.7, 1021.4, 678.0, and 399.3 cm^−1^ (see Figures 5 and 6). Using HD, absorptions are obtained at 1414.0 and 1412.5(sh) cm^−1^ between the two high‐frequency bands with H_2_, another absorption is obtained at 1030.0 cm^−1^ between the two high‐frequency bands with D_2_. The HD counterparts for the lower‐frequency absorptions are observed at 886.8 and 495.6 cm^−1^, between the corresponding H_2_ and D_2_ signals. In the present experiments, the signals previously assigned to VH_2_ and VH_2_(H_2_) in solid Ne^23^ have been observed weakly after irradiation.

### Assignments

The first set of bands is assigned to the linear HVVH molecule, the second set of bands is assigned to the folded V_2_(μ‐H)_2_ molecule with bridging H atoms. The assignment is based on the number of bands observed, the positions of the bands, and their isotopic shifts, and it is confirmed by computational results (density functional calculations with the TPSS functional and the TZVP basis set).

Within the first set of bands, when using H_2_, there is one band in the V−H stretching region at 1570.7 cm^−1^. (Note: for VH_2_ in Ne, the V−H stretching modes are found at 1492.9 and 1553.9 cm^−1^.^23^) When using D_2_, the band shifts to 1132.2 cm^−1^, confirming the presence of a V−H unit. Using HD, two bands in these regions at 1579.0 and 1138.6 cm^−1^ are found, one in the V−H and one in the V−D region, blue‐shifted with respect to their H_2_ or D_2_ counterparts. This indicates that the latter belong to pairs of bands, the higher‐lying member of which is not observed for symmetry reasons, pointing to a symmetrical arrangement of two V−H groups in accordance with linear HVVH. For linear HVVH, a total of two modes out of five (σ_u_^+^, π_u_) should be observable in infrared spectra, and indeed, another mode is observed at 343.4 cm^−1^, the ungerade combination of the bending motions. When using HD, due to symmetry reduction, yet another mode is observed at 442.8 cm^−1^, the formerly gerade combination (π_g_) of the bending vibrations. The density‐functional results nicely confirm the assignment (see below).

Within the second set of bands, when using H_2_, there are two signals at 1425.1 and 1405.3 cm^−1^ in the region of the stretching vibrations. They have lower wavenumbers than the signals assigned to HVVH, consistent with H in a bridging configuration. The isotopic shifts to 1035.7 and 1021.4 cm^−1^ when using D_2_ confirm that these bands are related to the motion of H atoms. The observation of two bands in the V−H stretching region already with the homonuclear reactants points to a non‐symmetric, likely non‐planar, structure. For folded *C*_2*v*_‐symmetric V_2_(μ‐H)_2_, five out of six modes (3×a_1_, b_1_, b_2_) should, in principle, be observable in infrared spectra. Two additional modes are observed at 917.2 and 565.4 cm^−1^, the shift of the H atoms along the direction of the V−V axis at 917.2 cm^−1^, the folding motion at 565.4 cm^−1^. Thus, out of the five in principle observable modes, four are found. The density‐functional calculations confirm the assignment to folded *C*_2*v*_ V_2_(μ‐H)_2_ and indicate that the other mode which, in principle, could be observed, is very weak, a mode dominated by the V−V stretching motion.

### Calculated Structures and Energies

**Figure 7:**
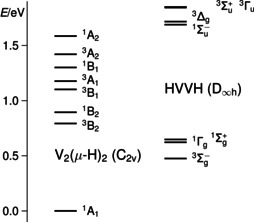
Relative energies of the low‐lying excited electronic states of folded V_2_(μ‐H)_2_ and linear HVVH at the structures of their ground states by Davidson‐corrected MRCI calculations with an ANO basis set.

The quantum‐chemical calculations, both MRCI and density‐functional calculations, yield as the lowest‐lying isomer of V_2_H_2_ a folded V_2_(μ‐H)_2_ isomer with a *C*_2*v*_‐symmetric structure, and, at an energy higher by about 0.5 eV, a linear HVVH isomer with *D*_∞*h*_ symmetry (Figure 7).

According to the MRCI calculations, the electronic ground state of the folded *C*_2*v*_ isomer is a ^1^A_1_ state. The V−V distance amounts to 184.4 pm and the V−H distance to 179.6 pm. The torsional angle between the two HV_2_ planes is 120.6°. The density‐functional calculations yield a broken‐symmetry state that corresponds to the ^1^A_1_ term. The values for the V−V and V−H distances of 182.9 and 180.1 pm, and the torsional angle between the HV_2_ planes of 113.3° obtained by the density‐functional calculations are close to the MRCI values and confirm that the density‐functional calculations give, qualitatively, the correct picture. The relative energies of low‐lying excited states (by MRCI) at the structure of the *C*_2*v*_^1^A_1_ ground term are shown in Figure 7. From a relative energy of 0.79 eV above the ground state, there are different triplet and singlet terms with small energy separations, the lowest ones are a ^3^B_2_ and a ^1^B_2_ term at 0.79 and 0.89 eV. The lowest‐lying term of the linear HVVH isomer is, according to the MRCI calculations, a ^3^Σ_g_^−^ term at an energy of 0.48 eV relative to the ^1^A_1_ state. The V−V distance amounts to 170.7 pm and the V−H distance to 170.5 pm. The same term is also obtained by the density‐functional calculations at a relative energy of 0.56 eV, in good agreement with the MRCI result. The values for the V−V and V−H distances of 171.1 and 171.6 pm obtained by the density‐functional calculations are again close to the MRCI values and confirm the qualitatively correct picture of the density‐functional calculations also in the case of the linear isomer. The relative energies of the lower‐lying terms at the structure of the ^3^Σ_g_^−^ term are also shown in Figure 7. At 0.15 and 0.17 eV with respect to the ^3^Σ_g_^−^ term, there are a ^1^Γ_g_ and a ^1^Σ_g_^+^ term, respectively. Further singlet and triplet terms follow at energies higher than 1.21 eV.

### Calculated Vibrational Frequencies

The vibrational frequencies obtained from the density‐functional calculations support the assignment of the observed bands to linear HVVH and folded V_2_(μ‐H)_2_. All vibrational modes for which the calculations indicate transitions with substantial intensity have been observed with wavenumbers not too far from the calculated values. For the linear isomer, the wavenumbers of the allowed transitions are calculated to be 1613 and 322 cm^−1^ (see Table 1), whereas the observed transitions have values of 1570 and 343 cm^−1^. The differences amount to 43 and 21 cm^−1^. The calculated isotopic shifts of 461 and 92 cm^−1^ show an even better agreement with the measured shifts of 438 and 97 cm^−1^. For the folded isomer, the calculated wavenumbers of 1522, 1502, 984, and 571 cm^−1^ (see Table 1) deviate from the measured values of 1425, 1405, 917 and 565 cm^−1^ by 97, 97, 66, and 5 cm^−1^, respectively. Thus, the observed splitting between the symmetric and antisymmetric vibrations at 1425 and 1405 cm^−1^ is almost quantitatively reproduced by the calculations. Again, the calculated isotopic shifts of 438, 431, 275, and 162 cm^−1^ still show a better agreement with the observed shifts of 389, 384, 239, and 166 cm^−1^. Such deviations of some 10 cm^−1^ are in the typical range for frequencies obtained by density‐functional calculations and have to be expected.

**Table 1:** Wavenumbers ($\tilde{\nu }$ /cm^−1^) of vibrational transitions of linear HVVH and folded V_2_(μ‐H)_2_ by density‐functional calculations with the TPSS functional and from IR spectra of Ne matrices. The calculated values are unscaled harmonic vibrational frequencies. Experimental values for the most prominent site after annealing to 10 K. (Entries in parenthesis: calculated intensities).

	V_2_(μ‐H)_2_				V_2_(μ‐H)(μ‐D)				V_2_(μ‐D)_2_		
	TPSS		Exp.		TPSS		Exp.		TPSS		Exp.
a_1_	328.0	(5)		a′	326.9	(5)		a_1_	324.7	(3)	
a_1_	571.1	(63)	565.4	a′	496.3	(51)	495.6	a_1_	408.9	(36)	399.3
a_2_	910.0	(0)		a′′	678.4	(6)		a_2_	656.0	(0)	
b_1_	983.7	(35)	917.2	a′′	950.6	(20)	886.8	b_1_	708.2	(17)	677.9
a_1_	1502.0	(98)	1405.3	a′	1077.4	(68)	1030.0	a_1_	1070.8	(47)	1021.4
b_2_	1521.9	(171)	1425.1	a′	1512.0	(134)	1414.0	b_2_	1083.9	(87)	1035.7

	HVVH				HVVD				DVVD		
	TPSS		Exp.		TPSS		Exp.		TPSS		Exp.
π_u_	322.0	(333)	343.4	π	264.6	(213)	276.7	π_u_	230.0	(170)	246.9
π_g_	550.1	(0)		π	504.9	(38)	442.8	π_g_	437.1	(0)	
σ_g_^+^	731.0	(0)		σ^+^	727.5	(0.3)		σ_g_^+^	723.9	(0)	
σ_u_^+^	1613.3	(670)	1570.7	σ^+^	1154.1	(172)	1138.6	σ_u_^+^	1152.3	(342)	1132.6
σ_g_^+^	1618.2	(0)		σ^+^	1615.7	(334)	1579.0	σ_g_^+^	1155.9	(0)

### Electronic Configurations

For the ^1^A_1_ ground state of the folded V_2_(μ‐H)_2_ isomer, the occupation of the valence‐space natural orbitals of the MRCI wave function, (8a_1_)^1.97^(3b_2_)^1.96^(9a_1_)^1.85^(10a_1_)^1.81^(4b_2_)^1.77^ (11a_1_)^1.70^(8b_1_)^0.28^(3a_2_)^0.21^(9b_1_)^0.18^(10b_1_)^0.11^(12a_1_)^0.04^(4a_2_)^0.03^ (5b_2_)^0.02^(11b_1_)^0.01^, clearly shows the multiconfigurational character. The leading configuration in the expansion of the wave function is (8a_1_)^2^(3b_2_)^2^(9a_1_)^2^(10a_1_)^2^(4b_2_)^2^(11a_1_)^2^ with a coefficient of 0.77. The first two orbitals are orbitals with a leading contribution from the H 1s orbitals. They are V−H bonding orbitals, the first one mainly involving the V 4s orbitals, the second one involving the 3d orbitals. The following eight orbitals are essentially 3d orbitals pointing to a substantial amount of 3d–3d bonding. Four of them are bonding orbitals and four of them are the corresponding antibonding orbitals. From the occupation numbers of the natural orbitals, a bond order of 3.17 is obtained. To confirm this consideration, a natural‐bond orbital analysis has also been performed. For the bridging hydrogen atoms, it yields two three‐center H−V_2_ bonding orbitals occupied with 1.96 electrons each and corresponding antibonding orbitals occupied with 0.03 electrons each. For the vanadium–vanadium interaction, it yields four V−V bonding orbitals occupied with 1.85, 1.81, 1.77, and 1.70 electrons, and corresponding antibonding orbitals occupied with 0.25, 0.21, 0.19 and 0.10 electrons. These values amount to 7.13 electrons in bonding orbitals and 0.75 electrons in antibonding orbitals, yielding a value of 3.19 for the bond order of the V−V bond, close to the value obtained by consideration of just the natural orbitals. Thus, the value of larger than three is indicative of the presence of fourfold bonding in V_2_(μ‐H)_2_.

For the ^3^Σ_g_^−^ ground state of the linear HVVH isomer, the occupation of the natural orbitals for the MRCI wave function is (6σ_g_^+^)^1.97^(6σ_u_^+^)^1.96^(7σ_g_^+^)^1.89^(3π_u_)^1.86^(3π_u_)^1.86^(1δ_g_)^0.90^ (1δ_g_)^0.90^(3π_g_)^0.12^(3π_g_)^0.12^(1δ_u_)^0.11^(1δ_u_)^0.11^(7σ_u_^+^)^0.09^(8σ_g_^+^)^0.04^ (8σ_u_^+^)^0.02^. The leading configuration is (6σ_g_^+^)^2^(6σ_u_^+^)^2^(7σ_g_^+^)^2^ (3π_u_)^2^(3π_u_)^2^(1δ_g_)^1^(1δ_g_)^1^ with a coefficient of 0.83. The first two orbitals are V−H bonding orbitals with the main contributions from H 1s and V 4s orbitals; the remaining ones are V 3d orbitals. Hence, the σ, π, and δ orbitals contribute to the V−V bonding. From the occupation numbers of the natural orbitals, a bond order of 3.43 is obtained, still somewhat larger than for the isomer with the bridging hydrogen atoms. The triplet ground state results from the occupation of the two δ orbitals with two electrons. An NBO analysis as well as the Mayer bond order based on the density‐functional results confirm the conclusions from the evaluation of the MRCI wave function (see the Supporting Information).

To obtain an estimate for the reaction energy of the formation of V_2_(μ‐H)_2_ and the fragmentation of HVVH, additional density‐functional calculations were performed on V_2_(^3^Σ_g_^−^), VH(^5^Δ), and H_2_. According to the calculations, the formation of V_2_(μ‐H)_2_ via V_2_+H_2_→V_2_(μ‐H)_2_ is exotherm by 1.18 eV. Hence, the formation of the linear HVVH isomer is still exotherm by 0.62 eV. The ^5^Δ ground state for VH is in line with previous calculations.^27^, ^28^, ^29^, ^30^ The calculated fragmentation energy of HVVH by HVVH(^3^Σ_g_^−^)→2 VH(^5^Δ) amounts to 2.77 eV. This value is similar to the experimental value of 2.75 eV for the dissociation energy of V_2_ 31 and higher than the value of 1.53 eV for Cr_2_,^32^ and supports the presence of multiple bonding in HVVH.

### Discussion

For both HVVH and V_2_(μ‐H)_2_, clear, sharp signals are observed only after annealing (not after deposition or irradiation), pointing to the population of a few (well‐defined) matrix sites. For HVVH, the broader signals found after deposition or irradiation are clearly apparent (Figure 2). Such signals, however, are not obvious for V_2_(μ‐H)_2_. Nevertheless, upon close inspection of the spectra after deposition or irradiation, also for V_2_(μ‐H)_2_, broad absorption features somewhat red‐shifted to sharper signals can be perceived (Figure 3). Additionally, in the spectra after deposition or irradiation, no signals are detected that would indicate the formation of yet another different product. Therefore, it is assumed that V_2_(μ‐H)_2_ is also present in the matrices after annealing or irradiation, but that the signals are too broad to be clearly seen. Notice that the signals of V_2_(μ‐H)_2_ are, in general, weaker than those of HVVH: The strongest signal of V_2_(μ‐H)_2_ has only about one quarter of the intensity of the strongest signal of HVVH. Thus, the detection of broad signals is expected to be more difficult for V_2_(μ‐H)_2_.

The experiments show that H_2_ reacts with V_2_ by formation of both a linear HVVH isomer and a folded *C*_2*v*_ V_2_(μ‐H)_2_ isomer. Thus, it is likely that the reaction proceeds without or with only a small barrier. The folded isomer is the more stable one, nevertheless, the linear isomer is formed in substantial amount. This observation is consistent with the ground‐state properties of folded V_2_(μ‐H)_2_ and linear HVVH, as revealed by the calculations. HVVH has a ^3^Σ_g_^−^ ground state, the same as the diatomic V_2_. Thus, the formation of HVVH from V_2_ and H_2_ is a spin‐allowed process, whereas the formation of the folded V_2_(μ‐H)_2_ isomer, which has a ^1^A_1_ ground state, is spin‐forbidden although energetically favorable. Irradiation with UV light leads to the destruction of HVVH and almost exclusive formation of the more stable V_2_(μ‐H)_2_. Obviously, by irradiation with UV light, higher‐lying excited states are populated that allow an easy isomerization to the folded V_2_(μ‐H)_2_, for example, by change of the spin state due to spin–orbit coupling and subsequent isomerization or vice versa.

**Scheme 1:**
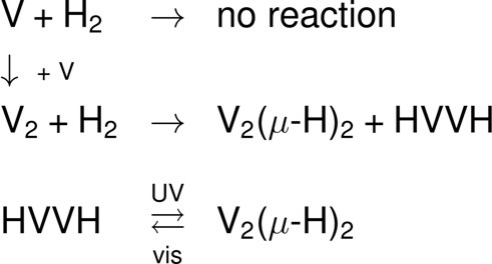
Formation of V_2_(μ‐H)_2_ and HVVH, and interconversion.

It is observed that upon irradiation with visible light, the signals of V_2_(μ‐H)_2_ decrease, whereas those of HVVH increase, and upon irradiation with UV light, the signals of HVVH almost entirely vanish, whereas those of V_2_(μ‐H)_2_ markedly increase. The experiments indicate the conversion of linear HVVH to non‐planar V_2_(μ‐H)_2_ by irradiation with UV light and the conversion of V_2_(μ‐H)_2_ to HVVH by irradiation with visible light (Scheme 1).

The chemistry of V_2_ and H_2_ is a reminiscence of the reactivity of the gallium dimer with dihydrogen. The gallium dimer Ga_2_, despite spin restrictions, spontaneously reacts with dihydrogen by formation of cyclic Ga_2_(μ‐H)_2_, whereas ground‐state Ga atoms do not react with H_2_. (GaH_2_ is formed only upon photoexcitation.)^33^, ^34^, ^35^ Furthermore, Ga_2_(μ‐H)_2_ features an interesting photochemistry. Irradiation (*λ*=546 nm) of Ga_2_(μ‐H)_2_ leads to the formation of HGaGaH and H_2_GaGa, and upon irradiation of these products using other wavelengths (*λ*=365 nm or *λ*>700 nm), cyclic Ga_2_(μ‐H)_2_ is reformed. Furthermore, the present results for V_2_H_2_ are similar to the findings of a theoretical (density‐functional) study on Ti_2_H_2_.^36^ There, also a folded isomer with bridging H atoms and a linear HTiTiH isomer were found, the isomer with bridging ligands being the lowest in energy.

The V−V bond distance of 170.7 pm for HVVH is surprisingly short, even shorter than that of V_2_(^3^Σ_g_^−^) of 180.4 pm,^37^ although the leading configuration for V_2_, (6σ_g_^+^)^2^(7σ_g_^+^)^2^(3π_u_)^2^(3π_u_)^2^(1δ_g_)^1^(1δ_g_)^1^, even points to a quintuple bond (The occupation numbers of the CASSCF natural orbitals for V_2_ yield a value for the bond order of 4.27). An explanation of this contradictory observation is likely the following: due to the formation of the V−H bonds, the 4s contribution to the electron density at the V atoms and in the V−V bonding region decreases, and therefore also the shielding of the V nuclear charge, and the V_2_ core has partial V_2_^2+^ character leading to a contraction of the V−V bonding orbitals. Although for both V_2_(μ‐H)_2_ and HVVH, the results point to quadruple bonding, the V−V bond distance in V_2_(μ‐H)_2_ is longer by 13.7 pm than in HVVH. The values for the V−V bond order are in qualitative agreement with this observation: the values point to quadruple bonding for both isomers, but the value for HVVH it is still somewhat larger than for V_2_(μ‐H)_2_. However, the bonding situation differs in some respects. In V_2_(μ‐H)_2_, there are bridging H atoms, and hence, there is associated electron density close to the V−V axis. Furthermore, HVVH has a triplet ground state. Therefore, in its leading configuration, two of the five orbitals that contribute to the bonding are occupied by one electron only, whereas in V_2_(μ‐H)_2_, there are four V−V bonding orbitals occupied by pairs of electrons. Thus, in HVVH, for the two unpaired electrons, there is the possibility to keep a larger distance. It is likely that both factors influence the final bonding situation.

The V−V bond distances of 184.4 pm and 170.7 pm in V_2_(μ‐H)_2_ and HVVH, respectively, are still shorter than the values in the paddlewheel complexes V_2_(DPhF)_4_ with a formal triple bond (197.8/197.9 pm)^3^ and K[V_2_(DPhF)_4_] with a formal bond order of 3.5 (192.9 pm),^4^ which have V_2_^4+^ and V_2_^3+^ cores, and far shorter than the value of 246.0 pm in a divanadium(IV) complex with a V−V single bond,^38^ supporting the presence of a quadruple bond in V_2_H_2_. HVVH and V_2_(μ‐H)_2_ are further examples of bimetallic metal hydride compounds with a high metal–metal bond order, like HCrCrH, HMoMoH, and HWWH.^39^ However, the latter ones with a *trans*‐bent alignment have been characterized only theoretically to date and are still awaiting experimental confirmation.

## Conclusion

Multiple bonds between two transition metals are intensely studied due to their intriguing electronic structure and potential applications. The bond order is particularly high if the number of ligands is decreased to an absolute minimum. However, the synthesis of RMMR compounds (M=transition metal) by conventional techniques in solution is a difficult task and, by all means, requires bulky ligands R. In this work, we demonstrate the use of the matrix‐isolation technique to experimentally characterize simple HMMH compounds with multiple MM bonds. Hence, two isomers of unprecedented V_2_H_2_, folded V_2_(μ‐H)_2_ and linear HVVH, are generated in solid Ne and identified by their IR spectra. UV irradiation induces the transformation of linear HVVH to folded V_2_(μ‐H)_2_. Irradiation with visible light leads to the transformation of the folded V_2_(μ‐H)_2_ isomer to linear HVVH. Quantum‐chemical calculations (MRCI) show that the folded V_2_(μ‐H)_2_ is the lower‐lying isomer with a ^1^A_1_ ground state. The linear HVVH with a ^3^Σ_g_^−^ electronic ground state is calculated to be at an energy of 0.48 eV with respect to the folded isomer. Both isomers are characterized by multiple V−V bonding interactions and the orbital occupations point to quadruple bonding. The calculated vibrational frequencies (density‐functional calculations) support the assignment of the observed transitions. Although the folded isomer is the lower‐lying one, without irradiation, the linear isomer is formed in a substantial amount. This observation is consistent with the electronic ground states of the isomers. Since the V_2_ molecule has a ^3^Σ_g_^−^ ground state, the formation of the folded V_2_(μ‐H)_2_ isomer (^1^A_1_) by reaction of H_2_ and V_2_ is a spin‐forbidden process, whereas the formation of linear HVVH is spin‐allowed. Thus, it is reasonable that a substantial amount is trapped as HVVH. In ongoing work, we extend our work on other multiple‐bonded HMMH compounds. The experimental characterization of these small molecules is important for the detailed understanding of the bond properties and, thereby, also the reactivity of these systems.

## Conflict of interest

The authors declare no conflict of interest.

## Supporting information

As a service to our authors and readers, this journal provides supporting information supplied by the authors. Such materials are peer reviewed and may be re‐organized for online delivery, but are not copy‐edited or typeset. Technical support issues arising from supporting information (other than missing files) should be addressed to the authors.

Supplementary
