# Supplementary material for: Oxidative Addition of Dihydrogen to Divanadium in Solid Ne: Multiple‐Bonded Triplet HVVH and Singlet V2(μ‐H)2
Source: Angew Chem Int Ed Engl. 2020 May 18;59(29):12206–12. doi: 10.1002/anie.202004241 (PMC7383563; doi:10.1002/anie.202004241)
Supplement: Supplementary file 1 — Supplementary [file ANIE-59-12206-s001.pdf]

Angewandte Chemie
Eine Zeitschrift der Gesellschaft Deutscher Chemiker

Supporting Information

Oxidative Addition of Dihydrogen to Divanadium in Solid Ne: Multiple-Bonded Triplet HVVH and Singlet $V_2(\mu\text{-}H)_2$

*Olaf Hübner and Hans-Jörg Himmel**

anie_202004241_sm_miscellaneous_information.pdf

1. Methods

The matrices are generated by codeposition of V atoms and mixtures of Ne (l'Air Liquide, 99.999 %) and H_2 (Messer, 99.999 %) onto a Rh-plated Cu surface cooled to 4.2 K by a pulse-tube refrigerator (Vericold). The H_2 content varies between 0.15 and 2 %. Experiments are also performed with D_2 (Messer, 99.7 %) and HD (Sigma-Aldrich 96 mol%). Vanadium is evaporated by resistively heating a 0.5 mm vanadium filament (Advent). During deposition the flux of the gas is held at 1.0 ml min^{-1} using a flow-controller (EL-FLOW, Bronkhorst). The deposition rate is monitored by a quartz microbalance and adjusted by controlling the electric current. Rates between 0.8 and 0.3 µg cm^{-2} min^{-1} are used. The matrices are annealed to 10 K and irradiated with visible (385-740 nm) or ultraviolet (250-385 nm) light using a Xe Lamp (Asahi Spectra). Absorption spectra are recorded with a Bruker Vertex 80v spectrometer. The spectra in the middle infrared range use a globar source, a KBr beam splitter and a mercury cadmium telluride detector. The spectra in the far infrared range use a Hg lamp source, a mylar multilayer beam splitter and a bolometer.
Density functional calculations are performed with the program Turbomole [1,2,3] and use the TPSS functional[4] and the def2-TZVP basis set.[5] We note that the ground state of the folded isomer of V_2H_2 obtained by the density functional calculations is a broken symmetry state with spin density of opposite sign localized at the two different V atoms, it is not a pure singlet state, but contains contaminations from states of higher spin multiplicity.
The multireference configuration interaction (MRCI) calculations are performed with the program system MOLPRO [6,7] in C_{2v} and D_{2h} symmetry relying on the internally contracted multireference configuration interaction program[8,9] and the multiconfigurational self-consistent field program.[10,11] The calculations use the 7s6p4d3f2g (V) and 4s3p2d (H) relativistic contractions of an atomic natural orbital (ANO) basis set.[12,13] Scalar relativistic contributions are included by means of the Douglas-Kroll-Hess formalism.[14] The orbitals for the multireference configuration interaction calculations are determined by complete active space self-consistent field (CASSCF) calculations. The active space of 14 orbitals contains the two 4s and the ten 3d orbitals of V and the two 1s orbitals of H. The MRCI calculations correlate the 4s, 3d, and 3p orbitals of V and the 1s orbitals of H. For the final energy determination, the reference space contains all configurations with a weight larger than 0.01 in the CASSCF wave function. The Davidson correction is applied. For the structure optimizations of HVVH a larger reference space selection criterion of 0.03 is used. Equilibrium distances are determined by a quasi-Newton algorithm.[15]
A natural bond orbital (NBO) analysis is performed for the results of the density functional calculations, using the Natural Bond Orbital program.[16] For this purpose, the (Kohn-Sham) orbitals of the ground states of HVVH and $V_2(\mu\text{-}H)_2$ are recalculated with the Gaussian program.[17] For the ground states of both isomers, also the Mayer bond order [18] is determined.

[1] TURBOMOLE V6.6 2014, a development of University of Karlsruhe and Forschungszentrum Karlsruhe GmbH, 1989-2007, TURBOMOLE GmbH, since 2007; available from http://www.turbomole.com.
[2] R. Ahlrichs, M. Bär, M. Häser, H. Horn, C. Kölmel, *Chem. Phys. Lett.* **1989**, *162*, 165-169.
[3] O. Treutler, R. Ahlrichs, *J. Chem. Phys.* **1995**, *102*, 346-354.
[4] J. Tao, J. P. Perdew, V. N. Staroverov, G. E. Scuseria, *Phys. Rev. Lett.* **2003**, *91*, 146401.

[5] F. Weigend, R. Ahlrichs, *Phys. Chem. Chem. Phys.* **2005**, *7*, 3297-3305.
[6] MOLPRO, version 2015.1, a package of ab initio programs, H.-J. Werner, P. J. Knowles, G. Knizia, F. R. Manby, M. Schütz, P. Celani, W. Györffy, D. Kats, T. Korona, R. Lindh, A. Mitrushenkov, G. Rauhut, K. R. Shamasundar, T. B. Adler, R. D. Amos, A. Bernhardsson, A. Berning, D. L. Cooper, M. J. O. Deegan, A. J. Dobbyn, F. Eckert, E. Goll, C. Hampel, A. Hesselmann, G. Hetzer, T. Hrenar, G. Jansen, C. Köppl, Y. Liu, A. W. Lloyd, R. A. Mata, A. J. May, S. J. McNicholas, W. Meyer, M. E. Mura, A. Nicklass, D. P. O'Neill, P. Palmieri, D. Peng, K. Pflüger, R. Pitzer, M. Reiher, T. Shiozaki, H. Stoll, A. J. Stone, R. Tarroni, T. Thorsteinsson, and M. Wang, see http://www.molpro.net.
[7] H.-J. Werner, P. J. Knowles, G. Knizia, F. R. Manby and M. Schütz, *WIREs Comput. Mol. Sci.* **2012**, *2*, 242–253.
[8] H.-J. Werner, P. J. Knowles, *J. Chem. Phys.* **1988**, *89*, 5803-5814.
[9] P.J. Knowles, H.-J. Werner, *Chem. Phys. Lett.* **1988**, *145*, 514-522.
[10] H.-J. Werner, P. J. Knowles, *J. Chem. Phys.* **1985**, *82*, 5053-5063.
[11] P. J. Knowles, H.-J. Werner, *Chem. Phys. Lett.* **1985**, *115*, 259-267.
[12] B. O. Roos, R. Lindh, P.-A. Malmqvist, V. Veryazov, P.-O. Widmark, *J. Phys. Chem. A* **2005**, *109*, 6575-6579.
[13] P.-O. Widmark, P.-A. Malmqvist, and B. O. Roos, *Theor. Chim. Acta* **1990**, *77*, 291-306.
[14] A. Wolf, M. Reiher, B. A. Hess, *J. Chem. Phys.* **2002**, *117*, 9215-9226.
[15] F. Eckert, P. Pulay, H.-J. Werner, *J. Comp. Chem.* **1997**, *18*, 1473-1483.
[16] NBO 6.0. E. D. Glendening, J. K. Badenhoop, A. E. Reed, J. E. Carpenter, J. A. Bohmann, C. M. Morales, C. R. Landis, and F. Weinhold (Theoretical Chemistry Institute, University of Wisconsin, Madison, WI, 2013); http://nbo6.chem.wisc.edu/
[17] Gaussian 09, Revision D.01, M. J. Frisch, G. W. Trucks, H. B. Schlegel, G. E. Scuseria, M. A. Robb, J. R. Cheeseman, G. Scalmani, V. Barone, G. A. Petersson, H. Nakatsuji, X. Li, M. Caricato, A. Marenich, J. Bloino, B. G. Janesko, R. Gomperts, B. Mennucci, H. P. Hratchian, J. V. Ortiz, A. F. Izmaylov, J. L. Sonnenberg, D. Williams-Young, F. Ding, F. Lipparini, F. Egidi, J. Goings, B. Peng, A. Petrone, T. Henderson, D. Ranasinghe, V. G. Zakrzewski, J. Gao, N. Rega, G. Zheng, W. Liang, M. Hada, M. Ehara, K. Toyota, R. Fukuda, J. Hasegawa, M. Ishida, T. Nakajima, Y. Honda, O. Kitao, H. Nakai, T. Vreven, K. Throssell, J. A. Montgomery, Jr., J. E. Peralta, F. Ogliaro, M. Bearpark, J. J. Heyd, E. Brothers, K. N. Kudin, V. N. Staroverov, T. Keith, R. Kobayashi, J. Normand, K. Raghavachari, A. Rendell, J. C. Burant, S. S. Iyengar, J. Tomasi, M. Cossi, J. M. Millam, M. Klene, C. Adamo, R. Cammi, J. W. Ochterski, R. L. Martin, K. Morokuma, O. Farkas, J. B. Foresman, and D. J. Fox, Gaussian, Inc., Wallingford CT, 2016.
[18] I. Mayer, *Chem. Phys. Lett.* **1983**, *97*, 270-274.

2. Bonding analysis

For $V_2(\mu\text{-}H)_2$, the results of an NBO analysis of the ground state density from the density functional calculations are shown in Table S1. The V-V bonding orbitals are occupied by altogether 7.914 electrons, and the corresponding antibonding orbitals by 0.126 electrons, yielding a bond order of 3.89 for the V-V bonding in $V_2(\mu\text{-}H)_2$. For the linear HVVH, the results of an NBO analysis of the ground state density from the density functional calculations are shown in Table S2. There are 7.998 electrons in V-V bonding orbitals and 0.019 electrons in the corresponding antibonding orbitals, yielding a bond order of 3.99 for the V-V bonding in HVVH. The NBO analysis of the density functional results also points to the presence of quadruple bonding in both $V_2(\mu\text{-}H)_2$ and HVVH and to a slightly higher bond order in HVVH compared to $V_2(\mu\text{-}H)_2$ and thus corroborates the results obtained from the consideration of the MRCI natural orbitals.

Table S1. Occupation numbers of the natural bond orbitals from a natural bond orbital analysis for the ground state density of $V_2(\mu\text{-}H)_2$ by density functional calculations with the TPSS functional and the def2-TZVP basis set. Abbreviations BD: 2-electron-2-center bonding orbital, 3C: 2-electron-3-center bonding orbital, BD*: 2-electron-2-center antibonding orbital, 3C*: 2-electron-3-center antibonding orbital, 3Cn: 2-electron-3-center non-bonding orbital.

Spin	Type	Atoms	Occupation	Spin	Type	Atoms	Occupation
alpha	BD	V1-V2	0.999	beta	BD	V1-V2	0.999
	BD	V1-V2	0.993		BD	V1-V2	0.993
	BD	V1-V2	0.985		BD	V1-V2	0.985
	BD	V1-V2	0.980		BD	V1-V2	0.980
	3C	V1-V2-H3	0.994		3C	V1-V2-H3	0.994
	3C	V1-V2-H4	0.994		3C	V1-V2-H4	0.994
	BD*	V1-V2	0.024		BD*	V1-V2	0.024
	BD*	V1-V2	0.021		BD*	V1-V2	0.021
	BD*	V1-V2	0.019		BD*	V1-V2	0.019
	BD*	V1-V2	0.009		BD*	V1-V2	0.009
	3Cn	V1-V2-H3	0.002		3Cn	V1-V2-H3	0.002
	3Cn	V1-V2-H4	0.002		3Cn	V1-V2-H4	0.002
	3C*	V1-V2-H3	0.001		3C*	V1-V2-H3	0.001
	3C*	V1-V2-H4	0.001		3C*	V1-V2-H4	0.001

Table S2. Occupation numbers of the natural bond orbitals from a natural bond orbital analysis for the ground state density of linear HVVH by density functional calculations with the TPSS functional and the def2-TZVP basis set. Abbreviations BD: 2-electron-2-center bonding orbital; BD*: 2-electron-2-center antibonding orbital.

Spin	Type	Atoms	Occupation	Spin	Type	Atoms	Occupation
alpha	BD	V1-V2	1.000	beta	BD	V1-V2	1.000
	BD	V1-V2	1.000		BD	V1-V2	1.000
	BD	V1-V2	1.000		BD	V1-V2	0.999
	BD	V1-V2	1.000				
	BD	V1-V2	0.999				
	BD	V1-H3	0.974		BD	V1-H3	0.980
	BD	V2-H4	0.974		BD	V2-H4	0.980
	BD*	V1-V2	0.006		BD*	V1-V2	0.005
	BD*	V1-V2	0.002		BD*	V1-V2	0.002
	BD*	V1-V2	0.002		BD*	V1-V2	0.002
	BD*	V1-V2	0.000				
	BD*	V1-V2	0.000				
	BD*	V1-H3	0.049		BD*	V1-H3	0.044
	BD*	V2-H4	0.049		BD*	V2-H4	0.044

The Mayer atomic bond orders have been determined based on the TPSS density functional results. For $V_2(\mu\text{-}H)_2$, the values obtained for the V-V and V-H bonds are 3.540 and 0.481, respectively. Thus, for the bridging H atoms, the values indicate the half of a bond to each of the V atoms, in accordance with the presence of 3-center bonds. For the linear HVVH, the values obtained for the V-V and V-H bonds are 3.601 and 1.113, respectively. Hence, the Mayer atomic bond orders indicate that within HVVH the V-V bond order is slightly larger than in $V_2(\mu\text{-}H)_2$.
